# Rituximab-Induced Interstitial Lung Disease: A Stitch in Time Saved Nine

**DOI:** 10.7759/cureus.27088

**Published:** 2022-07-20

**Authors:** Sai Tej, Asha Undrajavarapu, Kirti Kadian, Alkesh Khurana, Abhishek Goyal

**Affiliations:** 1 Pulmonary and Critical Care Medicine, All India Institute of Medical Sciences, Bhopal, Bhopal, IND

**Keywords:** ct scan, pemphigus, steroids, rituximab, ild

## Abstract

A 59-year-old male was initially diagnosed with pemphigus Vulgaris and received rituximab after a suboptimal clinical response with low-dose steroids and cyclophosphamide. Shortly after the third dose, he had acute interstitial pneumonia which was attributed to rituximab as there were no signs of any infective etiology after a detailed workup. He was put on mechanical ventilation but the dramatic response to pulse steroids helped the patient in early extubation and a favorable outcome.

## Introduction

Rituximab is a relatively newer immunosuppressive drug that acts against the CD20 antigen of the B cells and hence is used usually in disorders where B cells play a predominant role [1]. Common side effects of this drug include body aches, burning sensation on the skin, bloating of the face/arms, and blackish stool but pulmonary complications are extremely rare. This patient’s diagnosis of rituximab-induced interstitial lung disease (ILD) was that of exclusion and most importantly, needed enhanced immunosuppression (steroids) against an immunosuppressive drug itself. A favorable outcome prompts us to share this case with other medical professionals.

## Case presentation

**Figure 1:**
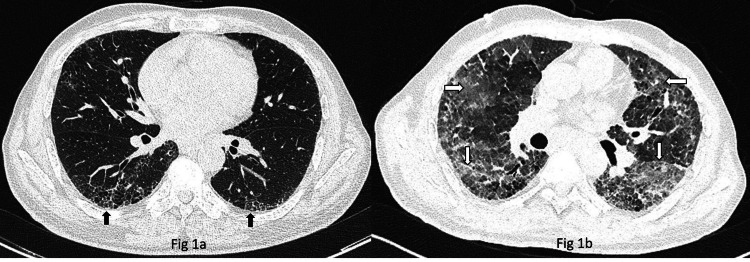
Rituximab-induced interstitial lung disease (a) Computed tomography scan of thorax shows bilateral basal reticulations and subtle honeycombing changes (upward arrows) consistent with a possibility of a usual interstitial pneumonia pattern. (b) Computed tomography scan of thorax shows extensive GGOs (horizontal arrows) and interlobular septal thickening (downward arrows) consistent with findings of acute interstitial pneumonia. GGOs - Ground Glass Opacities

A 59-year-old male was diagnosed with pemphigus Vulgaris in February 2022. The patient was initiated on low-dose steroids, cyclophosphamide 50 mg once a day initially by the dermatologist but because of progressive disease, he has escalated to rituximab 500 mg for three doses. Because of a history of previous treatment for pulmonary tuberculosis and occasional episodes of dry cough, a pulmonary consultation was sought and Computed Tomography (CT) scan of the thorax was advised as chest radiograph and pulmonary function tests were inconclusive. High-resolution CT revealed right-sided bronchiectasis and basal reticulations and subtle honeycombing changes (Figure 1a), which explained his symptoms. Autoimmune ILD, especially bullous lung disease has been reported in association with pemphigus but as there were no significant respiratory symptoms and dermatologists had already decided to start with immunosuppressants for a skin condition, he was advised no additional intervention from a pulmonary perspective. After a couple of days of receiving the third dose of rituximab the patient presented to the emergency with symptoms of fever, dry cough, and progressive shortness of breath (m-MRC 4). On examination, he was hypoxic with maintained SpO_2_ 92% with 8 liters of oxygen via face mask, pulse rate was 130/min, BP 130/80 mm of Hg, multiple discrete plaques few with crust present over face, chest, and back, and multiple hyperpigmented macules over the chest and back consistent with background diagnosis of pemphigus Vulgaris. On auscultation bilateral inframammary, infra-axillary, and infra-scapular fine inspiratory crepitations were present. A high-resolution CT scan was done and showed diffuse consolidation and Ground Glass Opacities (GGOs) with interlobar septal thickening in bilateral lung fields with honeycombing in bilateral lower lobes (Figure 1b). Considering the patient’s immunocompromised status, he was started on broad-spectrum antibiotics and Cotrimoxazole initially. The total leucocyte count was never beyond 11,000/mm^3^. Sputum, blood, and urine cultures were sterile, sputum CBNAAT - MTB was not detected, and urine microscopy was within normal limits. The patient’s oxygen requirement increased to 15 liters of oxygen and was not tolerating NIV, so the patient was eventually intubated and started on mechanical ventilation. Bronchoscopy was done which revealed no infection including acid-fast bacilli (AFB), aerobic, anaerobic, fungal, and viral organisms. Broncho alveolar lavage (BAL) fluid analysis though showed 90% lymphocytes. After thorough investigations, rituximab-induced ILD was suspected as a diagnosis of exclusion and temporal association of the symptoms/signs with the treatment of rituximab. After a detailed discussion, the patient was started on pulse steroids 500 mg methylprednisolone for three days followed by a 40 mg methylprednisolone maintenance dose. The patient improved significantly in terms of oxygen requirement and required pressure support and hence was extubated after four days of starting high-dose steroids which further supports the diagnosis of rituximab-ILD. The patient was discharged in a stable condition after two weeks of hospitalization but was lost to follow-up thereafter.

## Discussion

Rituximab is a chimeric G1-k monoclonal antibody channeled against a CD20 antigen present on the surface of B cells but not on plasma cells and stem cells. This makes it more selective towards the disorders caused by B cells and it also acts synergistically by accelerating apoptosis caused by antineoplastic drugs [1]. Pulmonary complications after rituximab are very rare and published in the literature mostly as sporadic case reports. These include organizing pneumonia (OP), acute respiratory distress syndrome (ARDS), acute interstitial pneumonia, and alveolar hemorrhage [2]. The mechanism behind the lung toxicity is unclear but it has been assumed to be due to the efficient clearing of the B cells both in the diseased organ and in the normal lung leads to the cascade of events releasing various proinflammatory mediators, cytokines and angiogenetic factors causing lung injury [3]. Liote et al. suggested that lung injury can be because by the hypersensitivity reaction to rituximab [2].

Most of the available literature has reported rituximab-induced ILD in patients without prior lung injury except for one case reported by Vulsteke et al., which reported worsening of underlying ILD with rituximab, almost similar to our case [4]. The case reported by Vulsteke et al. was an 87-year-old male with underlying diffuse large B-cell lymphoma and concomitant IPF. The case reported here also had background bilateral basal reticulations concomitant with pemphigus Vulgaris. The lung involvement in our case was not consistent with the ones reported in association with pemphigus (associated with bullous lung disease). Whether our patient suffered purely rituximab-induced new-onset ILD or was an exacerbation of preexisting ILD precipitated by rituximab remains a matter of discussion. Also, among the pattern of ILDs caused by rituximab mentioned above, OP remains the most steroid-responsive and our patient also did dramatically respond to steroids but the CT picture was far unrelated to OP. The lack of histopathology was a limitation in our case as the patient’s clinical condition did not give us a chance to get it done. Also, we could not get a follow-up CT for comparative assessment but the rapid clinical response to steroids was the obvious turning point in the management of this patient.

Diagnosis mostly rests on the temporal association of rituximab with the onset of symptoms/radiological findings. Because all patients receiving rituximab have already underlying immunosuppression, it is prudent to rule out an infectious etiology by doing all necessary investigations. CT findings include GGOs along with focal/diffuse alveolar infiltrates and rarely a macronodular pattern [2]. Our patient presented to ER after the third cycle of rituximab with symptoms of fever, dry cough, and SOB. The onset of pulmonary complications after the third or fourth dose of rituximab as happened in our case is not unusual and has been described by Liote et al. In the largest case series of 45 patients [2]. Krishnaswamy et al. also reported six patients of rituximab-induced ILD in patients of non-Hodgkin lymphoma whereby the median time of presentation was usually after the third dose and the presenting symptoms were acute onset of fever, cough, and SOB or its combinations [5].

Treatment of rituximab-induced ILD is supplement oxygen, methylprednisolone, and supportive ventilation [5,6]. The dosage and duration of steroids are not yet standardized as the cases encountered are quite rare. Our patient received high-dose steroids 500 mg methylprednisolone for three days followed by a maintenance dose after which the patient was extubated comfortably.

## Conclusions

In conclusion, rituximab-induced ILD is a rare but misdiagnosed and near fatal condition that can be treated with a high index of clinical suspicion. Because its symptoms and radiological findings are nonspecific, early diagnosis and treatment are the keys for this entity in preventing morbidity and mortality after ruling out infection which is the most common differential diagnosis.
